# Remdesivir: the first FDA-approved anti-COVID-19 Treatment for Young Children

**DOI:** 10.15190/d.2022.10

**Published:** 2022-06-30

**Authors:** Alexandra Chera, Antoanela Tanca

**Affiliations:** ^1^Carol Davila University of Medicine and Pharmacy, Bucharest, Romania; ^2^Victor Babes National Institute of Pathology, Bucharest, Romania

**Keywords:** COVID-19, young children, pediatric, remdesivir, benefits and limitations.

## Abstract

Following the emergence of the SARS-CoV-2 pandemic, finding efficient forms of treatment is seen as a priority for both adults and children. On April 25, 2022, remdesivir has become the first United States Food and Drug Administration (FDA) approved COVID-19 treatment for young children, specifically ≥28-days-old children, weighing ≥3 kilograms, who are either hospitalized or non-hospitalized, showing a high risk for progression to severe COVID-19 (prone to hospitalization or death). This new approval, which expands its already FDA-approved use in adults to young children, is supported by the CARAVAN study (a phase 2/3 single-arm, open-label study to evaluate the safety, tolerability, pharmacokinetics, and efficacy of remdesivir (GS-5734™) in participants, from birth to < 18 years of age, with COVID-19). This study is in progress, with an estimated primary completion in February 2023. While positive effects of remdesivir have been ascertained through various studies, controversy has surrounded remdesivir since its initial FDA approval in 2020 due to the contradictory results obtained by various studies. However, many case reports state its positive effects on the outcome of the patients, encouraging an optimistic vision for the future.

## SUMMARY

*1. Introduction*

*2.* *COVID-19 pathogenesis*

*3. SARS-CoV-2 infection and clinical manifestations in the pediatric population*

*4. Management of COVID-19 in pediatric patients*

*5. Remdesivir: a treatment option for children with COVID-19*

*6.* *Remdesivir: adverse reactions described in pediatric patients*

- * 6.1 Renal adverse reactions*

- * 6.2 Hepatic adverse reactions*

- * 6.3 Cardiovascular adverse reactions*

- *6.4 Other adverse events*

*7. Benefits and limitations*

*8. Conclusion*

## 1. Introduction

Severe acute respiratory syndrome coronavirus 2 (SARS-CoV-2), responsible for the coronavirus disease 2019 (COVID-19)^1^, has affected more than 505 million people worldwide. Data from April 2022 show at least 6 million deaths caused by SARS-CoV-2 infection^2^, with an increased number of cases in the pediatric population consecutive to the Omicron variant^3^. Even though the incidence of respiratory viral infections in children is generally high, SARS-CoV-2 has rather been associated with increased frequency and severity in adults, probably due to differences between the immune response^4^. However, children are prone to a particular form of disease known as Multisystem Inflammatory Syndrome in Children (MIS-C)^5^, which usually requires intensive care for those affected^4^. Many therapies have been tested for COVID-19, and some of them have shown a favorable response in patients – one of the drugs supported by evidence as treatment for SARS-CoV-2 infection is remdesivir (Veklury)^5^, proving itself beneficial in both adults and children. Remdesivir has been approved by the United States Food and Drug Administration (FDA) on April 25, 2022 as the first COVID-19 treatment for young children, specifically ≥28-days-old children, weighing ≥3 kilograms, who are either hospitalized or non-hospitalized, showing a high risk for progression to severe COVID-19 (prone to hospitalization or death)^6^. This came as an update on its FDA approval received on October 22, 2020, which included adults and ≥ 12-year-old children weighing ≥ 40 kilograms, all requiring hospitalization^7^.

## 2. COVID-19 pathogenesis

**Figure 1:**
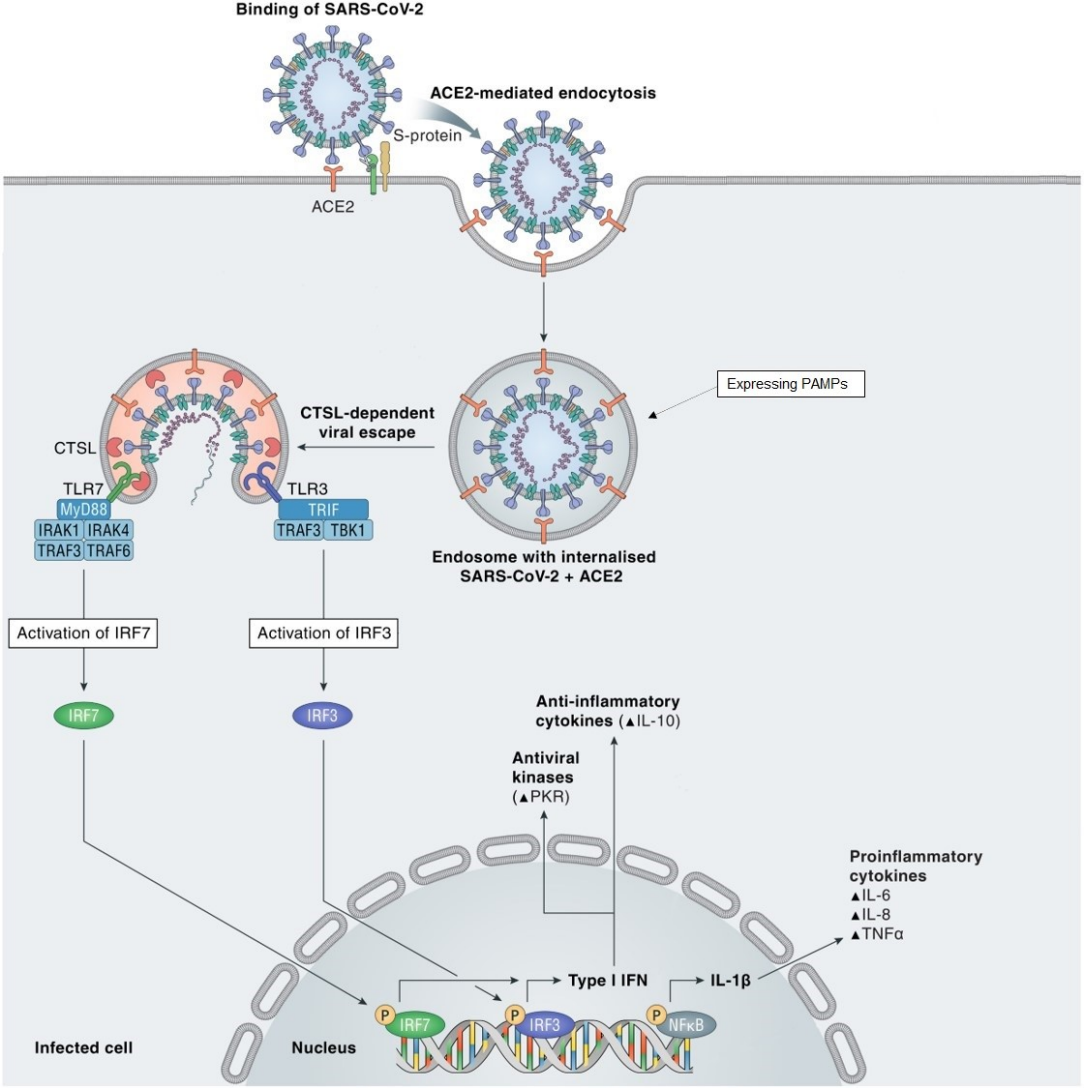
SARS-CoV-2 pathogenesis S protein binds to ACE2 and they enter the cell through endocytosis, followed by a process of cathepsin L (CTSL)-dependent viral escape from the endosome. PAMPs are intercepted by TLR3 and TLR7, which trigger intracellular signaling cascades by activating IRF3 and IRF7. This results in nuclear synthesis of cytokines such as type I IFN or IL-1β.Partially reproduced and adapted from reference^9^ (this is an open access article under the terms of the Creative Commons Attribution-NonCommercial-NoDerivs License, which permits use and distribution in any medium, provided the original work is properly cited^9^).

SARS-CoV-2 belongs to the beta genus of the *Coronaviridae* family, order *Nidovirales*. Coronaviruses have a particular structure which includes 4 types of proteins: spike (S), membrane (M), envelope (E), and nucleocapsid (N) proteins. The most important for COVID-19 pathogenesis is the S protein, as it targets the highly expressed angiotensin-converting enzyme 2 (ACE2) in lungs, gastrointestinal tract, heart, blood vessels, kidneys^8^. When the S protein binds to ACE2, the virus expressing viral pathogen-associated molecular patterns (PAMPs) enters the cytoplasm via endocytosis, where Toll-like receptors (TLRs) are able to recognize the PAMPs. Following this process, intracellular signaling cascades are initiated, leading to type I interferons (IFNs) and pro-inflammatory cytokines production (see Figure 1)^9^. 

Two steps have been described in COVID-19 pathogenesis: firstly, the virus triggers the initial respiratory symptoms and it is either eliminated at the end of this step, or it aggravates the lung affliction. Secondly, in some patients a so-called ‘cytokine storm’ may occur, leading to hyperinflammation. This causes acute respiratory distress syndrome (ARDS) with respiratory failure in adults and MIS-C with reduced lung affliction in children^4^. Several physiopathological mechanisms responsible for COVID-19 progression are: virus-induced cytopathic effects, renin–angiotensin–aldosterone system imbalance due to ACE2 downregulation, the ‘cytokine storm’ caused by an abnormal immune response, coagulopathy, thrombotic microangiopathy and autoimmunity^9^.

## 3. SARS-CoV-2 infection and clinical manifestations in the pediatric population

18.9% of all cases of COVID-19 are found in pediatric patients: 12,042,870 cases of SARS-CoV-2 infection in children were described on February 3, 2022 in the United States of America (USA)^3^. In the pediatric population, SARS-CoV-2 is transmitted through droplets or aerosols, usually from positive adults, and it is characterized by a variable viral incubation period of 2-10 days. Other transmission routes mentioned in literature are the fecal-oral route^8^, vertical transmission (due to the high expression of ACE2 in the placenta), as well as transmission through human milk, which has not been thoroughly confirmed yet^10^. There are many risk factors involved in disease progression, such as race (higher risk in African-American and Hispanic children), gender (males are more inclined to suffer from severe forms of COVID-19)^11^ and comorbidities, which can be stratified by age group in two categories: < 2-year-olds, usually prematurely born with chronic lung disease, airway malformations, neurologic and cardiovascular disease, and 2 to 17-year-old individuals with obesity, diabetes mellitus and feeding tube dependency^3^. Regarding respiratory disease, asthma and cystic fibrosis are associated with mild forms of COVID-19, while bronchodysplasia aggravates the prognosis^4^. Other comorbidities associated with increased demand of pediatric intensive care unit admission are genetic anomalies, malignancy, immune suppression, chronic kidney and liver disease, malnutrition, as well as sickle cell anemia^8^. The aforementioned comorbidities designate the so-called “children with medical complexity”, which bear the risk of increased severity of COVID-19 through decompensation of the chronic disease^12^. There is contradictory evidence regarding age as a risk factor for increased severity of the disease: some data indicate that infants younger than 6 months have a higher risk of severe disease than older children^12^, while other studies pin the increased illness severity on older children. Fortunately, the mortality of SARS-CoV-2 infected children admitted into the intensive care unit is significantly lower than mortality found in adults^13^. This might be due to the fact that ACE2 receptor in alveolar type 2 cells is different in children, or because of the immune system particularities found in the pediatric population. Also, the lower rate of some comorbidities (arterial hypertension, heart disease, type 2 diabetes) in children should not be overlooked. COVID-19 has five different clinical presentations in pediatric cases – asymptomatic, mild, moderate (developing mild respiratory distress), with severe respiratory symptoms and requiring intensive care unit admission (respiratory failure or MIS-C)^4^. Alongside the respiratory symptoms, digestive symptoms may occur rather frequently (diarrhea, abdominal pain, vomiting), and less often dermatologic lesions (urticarial or vesicular eruptions, livedo reticularis, pernio-like acral lesions) might be present^8^. The severity of COVID-19 in children has not been internationally classified. The guideline regarding the administration of remdesivir defines a severe case by its supplemental oxygen requirement and need for medical ventilation^14^. MIS-C itself is described as a post-infectious inflammatory phenomenon, even though it can also appear while the viral infection is active^12^.The immune response responsible for MIS-C is similar to the one found in Kawasaki disease. Its symptoms include: fever, systemic inflammatory syndrome and multiple organ dysfunction^15^.

## 4. Management of COVID-19 in pediatric patients

Since randomized control trials usually include adults, the clinical practice guidelines regarding COVID-19 management in children are mostly based on extrapolations of the results attained for the adult population^2^. COVID-19 cases in the pediatric population are usually less severe than those found in adults, therefore supportive care is often satisfactory for both neonates^16^ and older children. However, antiviral therapy with remdesivir is a viable option for patients with severe forms of the disease^13^. The first step in case management consists of testing the patients with increased suspicion of SARS-CoV-2 infection, using reverse transcription polymerase chain reaction (RT-PCR) as method of diagnosis. In neonates, testing is recommended 12-24 hours after birth, with a second test performed 24 hours after the first one. There are three scenarios found in neonates: congenital infection in live born neonates (SARS-CoV-2 infection confirmed in mother and child using RT-PCR for viral ribonucleic acid (RNA) detection in umbilical cord blood, neonatal blood collected within the first 12 hours after birth, or amniotic fluid), neonatal infection acquired intrapartum (SARS-CoV-2 infection confirmed in mother and child using RT-PCR for viral RNA detection in nasopharyngeal swab within the first 24-48 hours after birth) and neonatal infection acquired postpartum (SARS-CoV-2 infection confirmed in the ≥ 2 days-old child, with/without infection present in parent)^10^. Supportive care for COVID-19 pediatric patients includes respiratory support, maintaining fluid and electrolyte balance, suitable nutrition, second infection prophylaxis and extracorporeal membrane oxygenation. Symptomatic treatment is also provided^8^. Regarding antiviral treatment, remdesivir is the only FDA approved drug for young children (since April 25, 2022), in both hospitalized and non-hospitalized patients who are considered to be at high risk for complications – this category of children might include those suffering from diabetes mellitus, especially in association with obesity^12^ (which, itself, might increase the probability of hospitalization)^14^, and also patients diagnosed with chronic pulmonary disease^17^. High intensity therapies should be avoided when treating oncologic pediatric patients^18^. Other drugs that could be used for treating SARS-CoV-2 patients and/or its induced symptoms in patients are dexamethasone (for patients with respiratory distress requiring oxygen or ventilatory support), bamlanivimab, casirivimab or imdevimab (monoclonal anti-SARS-CoV-2 S protein antibodies used within complicated cases of COVID-19), tocilizumab and anakinra (when the patient has contraindications for administering remdesivir)^19^.

## 5. Remdesivir: a COVID-19 treatment option for children

Remdesivir (Veklury) is a prodrug that belongs to the class of phosphoramidate compounds. Its antiviral activity is accomplished by conversion to the active metabolite GS-443902 triphosphate (also known as GS-441524)^20^, which is able to incorporate itself into viral RNA, leading to premature termination^21^. It is an adenine nucleotide analogue^20^ developed in 2017 for treating Ebola virus infection^5^, but not being able to treat this disease successfully. However, its safety has made it a candidate for drug repurposing research^22^. It has broad-spectrum antiviral activity against Filoviruses (e.g., Ebola virus), Paramyxoviruses (e.g., respiratory syncytial virus) and Coronaviruses (including SARS-CoV-2)^23^. Remdesivir inhibits an enzyme called RNA-dependent RNA polymerase (RdRp) which is required for SARS-CoV-2 replication inside the host cells. Therefore, the viral RNA replication is interrupted after remdesivir administration. There is also another antiviral mechanism mentioned, which involves another enzyme - main protease (Mpro), responsible for cleaving polyproteins and coordinating SARS-CoV-2 replication. Remdesivir and its active metabolite bind to Mpro, exerting an additional inhibition of viral replication^24^. After successful administration of remdesivir to the first COVID-19 patient (confirmed on January 20, 2020), the symptom resolution and the favorable outcome of the patient have served as proof of its beneficial effects on treating SARS-CoV-2 infection^25^. Subsequently, FDA has launched a Coronavirus Treatment Acceleration Program in order to develop eligible therapeutic options for treating SARS-CoV-2 infection^26^. On May 1, 2020, FDA granted an emergency use authorization for remdesivir administration in severe cases of COVID-19, for both adults and children^5^. Shortly after, the evidence regarding the beneficial effects of remdesivir have been strengthened by new studies^12^, leading to expanding the approval for administration to non-severe cases of COVID-19 as well, on August 28, 2020^24^. On October 22, 2020, remdesivir became the first FDA approved drug for treating COVID-19 in ≥ 12 years-old children and adolescents, weighing at least 40 kg and in need of hospitalization, with an additional approval for emergency use in hospitalized pediatric patients weighing ≥ 3.5 kg, that were either < 12 years-old or weighted <40 kg^15^. Remdesivir use in hospitalized children has increased significantly after October 2020, as it has been administered to almost 60% of hospitalized children during November and December^27^. Remdesivir has become the first FDA approved COVID-19 treatment for children younger than 12 years-old on April 25, 2022. The specific guidelines include using injectable remdesivir for ≥ 28 days-old children, weighing ≥ 3 kilograms, who are either hospitalized or non-hospitalized but showing a high risk for progression to severe COVID-19 (prone to hospitalization or death)^6^. The new FDA approval is supported by the CARAVAN Study (a phase 2/3 single-arm, open-label study to evaluate the safety, tolerability, pharmacokinetics, and efficacy of remdesivir (GS-5734™) in participants from birth to < 18 years of age with COVID-19), with an estimated primary completion in February 2023, which includes 8 cohorts of pediatric participants (62 enrolled – May 13, 2022). 5 of them include children ≥ 28 days to < 18 years-old, 2 cohorts include 0-28 days-old term neonatal participants, and there is also a cohort of preterm neonates and 0-56 days-old infants. All of them receive age-appropriate doses of remdesivir for up to 10 days^28^ (see Table 1).

**Table 1:** CARAVAN study cohorts and remdesivir treatment administered to each cohort according to age and weight^28^.

Cohort	Age and weight	Remdesivir dosage
Cohort 1	12-18 years-old weighing ≥ 40 kg	Intravenous remdesivir 200mg (loading dose, on day 1) followed by 100 mg/day
Cohort 2	≥ 28 days-old to < 18 years-old weighing 20-40 kg	Intravenous remdesivir 5mg/kg (loading dose, on day 1) followed by 2.5 mg/kg/day
Cohort 3	≥ 28 days-old to < 18 years-old weighing 12-20 kg	Intravenous remdesivir 5mg/kg (loading dose, on day 1) followed by 2.5 mg/kg/day
Cohort 4	≥ 28 days-old to < 18 years-old weighing 3-12 kg	Intravenous remdesivir 5mg/kg (loading dose, on day 1) followed by 2.5 mg/kg/day
Cohort 5	14-28 days-old, gestational age > 37 weeks and weight at screening ≥ 2.5 kg	Intravenous remdesivir 5mg/kg (loading dose, on day 1) followed by 2.5 mg/kg/day
Cohort 6	0-14 days-old, gestational age > 37 weeks and birth weight ≥ 2.5 kg	Intravenous remdesivir 2.5mg/kg (loading dose, on day 1) followed by 1.25 mg/kg/day
Cohort 7	0-56 days-old, gestational age ≤ 37 weeks and birth weight ≥ 1.5 kg	Intravenous remdesivir 2.5mg/kg (loading dose, on day 1) followed by 1.25 mg/kg/day
Cohort 8	< 12 years-old weighing ≥ 40 kg	Intravenous remdesivir 200mg (loading dose, on day 1) followed by 100 mg/day

In addition, many case reports revealing remdesivir usage with a favorable outcome in the pediatric population have been cited in the literature (see Table 2).

**Table 2:** A series of cases cited from literature showing different approaches and outcomes of remdesivir treatment in the pediatric population **Abbreviations:** weeks of gestation (WoG); day of hospitalization (DoH); Continuous Positive Airway Pressure (CPAP); Positive end-expiratory pressure (PEEP); postinfectious bronchiolitis obliterans (PIBO); respiratory syncytial virus (RSV); body mass index (BMI); alanine aminotransferase (ALT); acute lymphoblastic leukemia (ALL); atrioventricular valve regurgitation (AVVR); pro–brain natriuretic peptide (BNP); aspartate aminotransferase (AST).

Citation	Patients treated with remdesivir	Comorbidities/Patient particularities	Treatment	Remdesivir Treatment Duration	Outcomes	Adverse drug reactions (ADR) to remdesivir
Hopwood AJ et al., 2020^29^	1, neonate [birth - 40 weeks of gestation (WoG)]	-mother: 15-year-old, tested positive for SARS-CoV-2 at birth -patient tested positive for SARS-CoV-2 24 hours after birth (presumed vertical transmission)	-supplemental oxygen; continuous positive airway pressure (CPAP) [started on the 4th day of hospitalization (DoH)] -remdesivir 5mg/kg (loading dose), followed by 2.5 mg/kg/day (DoH 4-13) -dexamethasone 0.15mg/kg/day (DoH 5-8) -positive end-expiratory pressure (PEEP); convalescent COVID-19 plasma: first dose of 10mL/kg, then 15mL/kg 24 hours later (DoH 8) -vancomycin (DoH 19-28)	10 days	-favorable outcome, after 13 days of intubation and ventilation, and a total of 30 days of CPAP use.	Bradycardia (on DoH 6)
Sarhan MA et al., 2022^16^	1, neonate (birth – 34 WoG)	-mother: 34-year-old, tested positive for SARS-CoV-2 4 hours before birth -patient tested positive for SARS-CoV-2 24 hours after birth (presumed vertical transmission)	-intubation; ventilatory support; dexamethasone 0.15mg/kg/day (DoH 6) -midazolam, morphine, rocuronium (DoH 8) -high dose of dexamethasone (0.5mg/kg/day) (DoH 8) -remdesivir 2.5mg/kg (loading dose), followed by 1.25 mg/kg/day (DoH 8-13)	6 days	-favorable outcome, the respiratory status improved within 2 days of remdesivir; the patient was off all respiratory support on DoH 25, and was discharged home on DoH 31.	None
Frauenfelder C et al., 2020^30^	1, 5 weeks after birth (birth - 32+6 corrected WoG)	-ex-premature twin -maternal preeclampsia -small atrial septal defect (<4mm), cleft palate	-ventilatory support -remdesivir 5 mg/kg (loading dose), followed by 1.25 mg/kg/day	10 days	-favorable outcome, the patient tested negative for SARS-CoV-2 after 5 days of remdesivir, was extubated on DoH 18, and was discharged home.	None
Saikia B et al., 2021^31^	2: 1st: 1 week after birth (birth – 31 WoG) 2nd: 35+2 corrected weeks (birth – 33 WoG)	-ex-premature patients	1st: -cefotaxime, amoxicillin, gentamicin, acyclovir (empirically) -respiratory support (DoH 6) -hydrocortisone 0.5mg/kg/day (DoH 7-16) -remdesivir 2.5mg/kg (loading dose), followed by 1.25mg/kg/day (DoH 11-15) 2nd: -cefotaxime, amoxicillin, gentamicin, acyclovir (empirically) -intubation shortly after admission -remdesivir 2.5mg/kg (loading dose), followed by 1.25mg/kg/day (DoH 4-7) -dexamethasone 150µg/kg/day (DoH 4-10)	1st: 5 days 2nd: 4 days	1st: -favorable outcome, patient was extubated after 2 days of remdesivir and had a negative result for SARS-CoV-2 infection after completing the treatment with remdesivir. 2nd: -favorable outcome, the patient and came off oxygen on DoH 5, had a negative result for SARS-CoV-2 infection after 2 days of remdesivir, and was discharged home on DoH 9.	None
Cursi L et al., 2021^32^	1, 9-day-old (birth – 36 WoG)	-ex-premature -cerebral venous thrombosis (as a complication of COVID-19)	-anti SARS-CoV-2 hyperimmune plasma -dexamethasone 0.15mg/kg/day -remdesivir 2.5mg/kg (loading dose), followed by 1.25mg/kg/day (started on day 5) -enoxaparin 100 UI/kg	10 days	-favorable outcome, the patient was extubated after 13 days and became oxygen independent after 4 more days.	None
Koletsi P et al., 2021^17^	1, 3-year-old	-severe postinfectious bronchiolitis obliterans (PIBO), diagnosed at 13-months-old after an episode of respiratory syncytial virus (RSV) and adenovirus coinfection -mixed hypercapnic and hypoxemic respiratory failure	-broad spectrum antibiotics -dexamethasone 0.15mg/kg/day (DoH 1-5) -remdesivir 5mg/kg (loading dose), followed by 2.5mg/kg/day (DoH 4-8)	5 days	-favorable outcome, with symptom regression 24 hours after the first dose of remdesivir; the patient was discharged home on DoH 11.	None
Jo Y et al., 2021^14^	1, 9-year-old	-obesity [body mass index (BMI) of 27.6kg/m²]	-oxygen therapy (since DoH 1) -dexamethasone 6mg (0.15mg/kg, with a maximum dose of 6mg) (DoH 2-11) -remdesivir 200mg (loading dose for body weights ≥ 40 kg), followed by 100mg/day (DoH 2-6)	5 days	-favorable outcome, with symptom regression on DoH 4; the patient came off oxygen on DoH 9 and was discharged home on DoH 21.	None
Chow EJ et al., 2021^33^	1, 16-year-old	-obesity (BMI of 43.9kg/m²)	-oxygen therapy (since DoH 1) -remdesivir 200mg (loading dose)	1 day (treatment stopped after the loading dose due to ADR)	-the patient had a favorable outcome despite stopping the remdesivir treatment after only one dose (due to the presence of bradycardia). The heart rates increased and the patient returned to the usual state of health in the week after hospital discharge.	Reversible sinus bradycardia: within 6 hours after remdesivir administration, the lowest heart rate was 46 bpm.
Eleftheriou I et al., 2021^34^	3: 1st: 13.5-year-old 2nd: 10-year-old 3rd: 3-month-old	None	-ampicillin -remdesivir -dexamethasone	-1st: 5 days -2nd: 5 days -3rd: 3 days (treatment stopped after the occurrence of ADR)	-heart rates returned to normal after completing the treatment for the 1st and 2nd patient, and 24 hours after discontinuation of remdesivir in the 3rd.	Asymptomatic sinus bradycardia: after administering the third (2nd and 3rd patient) or fourth dose (1st patient) of remdesivir.
Sanchez-Codez MI et al., 2021^35^	1, 13-year-old	-asthma	-oxygen therapy -dexamethasone -ceftriaxone -remdesivir 200mg (loading dose), followed by 100mg/day	3 days (treatment stopped after the occurrence of ADR)	-heart rate returned to normal 24 hours after discontinuation of remdesivir treatment	Asymptomatic sinus bradycardia: after the third dose of remdesivir (heart rate of 40 bpm)
Wardell H et al., 2020^36^	1, 19-day-old	-suspicion of evolving myocardial injury [elevated high sensitivity troponin T and N-terminal portion of pro–brain natriuretic peptide (BNP); mildly depressed left ventricular function]	-remdesivir 5mg/kg (loading dose), followed by 2.5mg/kg/day (started on DoH 4) -enoxaparin 0.8mg/kg/day (on DoH 5) -aspirin 20.25mg/day (on DoH 6)	7 days	-favorable outcome, patient was discharged on DoH 9; the systolic function and ejection fraction were normal 3 weeks after discharge.	None
Faltin K et al., 2022^37^	1, 17-year-old	-Friedrich’s ataxia-induced hypertrophic cardiomyopathy -atrial fibrillation, heart failure	-remdesivir 200mg (loading dose), followed by 100mg/day -convalescent plasma (anti-SARS-CoV-2 titer 1:600; 1 unit of 200mL)	5 days	-favorable outcome, with normalization of myocardial injury markers subsequent to remdesivir administration; the patient was discharged on DoH 17.	None
Orf K et al., 2020^38^	1, 5-year-old	-precursor B-cell acute lymphoblastic leukemia	-nebulized adrenalin -a single dose of oral dexamethasone -remdesivir 5mg/kg (loading dose), followed by 2.5mg/kg/day (started on DoH 2) -ALL induction chemotherapy (dexamethasone, vincristine, pegylated asparaginase) (started on DoH 2)	5 days (treatment stopped after the occurrence of ADR)	-favorable outcome (regarding COVID-19), the patient was discharged on day 8 of chemotherapy induction	-increased alanine aminotransferase (ALT) (maximum value: 408 U/L) on DoH 5, which returned to normal after interrupting remdesivir administration (within 10 days)
Dell’Isola GB et al., 2021^18^	1, 6-year-old	-acute lymphoblastic leukemia (ALL)	-remdesivir 5mg/kg (loading dose), followed by 2.5mg/kg/day -convalescent plasma -antileukemic treatment	5 days	-favorable outcome (regarding COVID-19), a rapid resolution of the infection was obtained.	None
Gadzińska J et al., 2022^39^	1, (nearly) 10-year-old	-ALL	-antileukemic treatment -oxygen therapy (DoH 23) -remdesivir 5mg/kg (loading dose), followed by 2.5mg/kg/day (DoH 26-30)	5 days	-favorable outcome (regarding COVID-19), the patient was discharged home on DoH 38.	None
Rodriguez Z et al., 2020^40^	1, 9-week-old	-trisomy 21 -unrepaired balanced complete atrioventricular canal defect -mild to moderate atrioventricular valve regurgitation (AVVR)	-bilevel noninvasive positive pressure ventilation (DoH 12) -remdesivir 5mg/kg (loading dose), followed by 2.5mg/kg/day (DoH 15-25) -convalescent plasma	11 days	-there was a lack of response to remdesivir, followed by deterioration of the patient’s clinical status despite 11 days of treatment.	None
Patel PA et al., 2020^41^	1, 12-year-old	-severe thrombocytopenia (presumably associated with the severe form of COVID-19)	-immunoglobulin -steroids -mechanical ventilation, inhaled nitric oxide, airway pressure release ventilation -azithromycin -hydroxychloroquine 400mg twice daily on DoH 4 followed by 200mg twice daily until DoH 7 -tocilizumab 8mg/kg, 2 doses, 12 hours apart) -remdesivir 200mg (loading dose) followed by 100mg/day (started on DoH 7)	6 days (treatment stopped after the occurrence of ADR)	-favorable outcome, even though remdesivir was discontinued, the patient was extubated on DoH 14 and discharged on DoH 24	-mildly elevated transaminases, which led to interrupting remdesivir treatment on DoH 12.
Kaur M et al., 2022^42^	1, 14-day-old	None	-CPAP -intubation (DoH 5) -remdesivir 5mg/kg (loading dose) (DoH 5)	1 day (treatment stopped after the loading dose due to ADR)	- after interrupting the remdesivir treatment, the liver enzymes decreased to normal ranges until DoH 15.	-significant transaminase elevation [maximum value for aspartate aminotransferase (AST): 1121 U/L and maximum value for ALT: 832 U/L)

Since the bioavailability by oral administration is not adequate, remdesivir can only be administered intravenously and it has a half-time of 1 hour, while its active metabolite has a longer half-time of 40 hours. The drug is metabolized within plasma and liver; thus, it is contraindicated in patients with liver disease, and while its renal excretion is low, there is one metabolite (GS-441524) that is excreted renally in a higher quantity, making remdesivir contraindicated in patients with impaired renal function. However, these data are obtained through adult studies and extrapolated into the pediatric population^19^. Testing the liver and kidney functions prior to remdesivir administration is mandatory^4^.

Drug interactions need to be analyzed, as they have multiple implications in the patient management. Remdesivir is a weak inhibitor of cytochrome P450 3A4 (CYP3A4), organic anion transporter protein 1B1 (OATP1B1), organic anion transporter protein 1B3 OATP1B3, and Multidrug and Toxin Extrusion Protein 1 (MATE1) in vitro, but studies have shown that its inhibitory actions on drug-metabolizing enzymes or transporters are not clinically significant in COVID-19 patients treated with therapeutic doses of remdesivir^23^. Observational studies on adult populations revealed that clinical benefits could be obtained while using dexamethasone in addition to remdesivir treatment^3^. Associations with azithromycin and antiepileptic drugs should be avoided because of the risk of hepatotoxicity^4^, and also the latter can be responsible for reducing remdesivir serum concentration by activating CYP3A4 and cytochrome P450 family 2 subfamily C member 9 (CYP2C9). It has been proved that Hydroxychloroquine has an antagonistic effect on the antiviral effect linked to remdesivir.

Therefore, the two should not be used together for treating COVID-19^43^.

## 6. Remdesivir: Adverse Drug Effects described in pediatric patients

Similar to every other medicine, adverse drug effects of remdesivir should be noted and taken into consideration when selecting it as treatment for a patient. It is known that its active metabolite accumulates in kidneys, liver and digestive tract^24^; thus, its most frequent adverse drug effects are kidney injury and hepatic enzymes elevation^44^, which can ultimately increase COVID-19 severity in patients with pre-existing complications^45^ Remdesivir use could be hazardous for patients with ALT or AST levels that are 5 times higher than the upper limit of normal, patients with the estimated glomerular filtration rate (eGFR) <30 mL/min, as well as pregnant or lactating women^24^.

### 6.1 Renal adverse drug reactions

Acute kidney injury (AKI) is quite common in COVID-19 patients and it involves glomerular, tubular and vascular affliction in the kidneys^9^. Unfortunately, remdesivir itself can further worsen the kidney injury as its metabolite (GS-441,524) can be found in urine after drug intake^44^, but also through kidney accumulation of an excipient (sulfobutylated beta-cyclodextrin sodium salt), especially in pediatric patients with previous renal impairment which receive renal replacement therapies.

Therefore, remdesivir is not recommended in children of ≥28-days-old with an eGFR <30mL/min and full-term neonates (<28-days-old) with a serum creatinine of ≥ 1mg/dL^43^.

### 6.2 Hepatic adverse drug reactions

Elevation of liver transaminases and hyperbilirubinemia can be found in COVID-19 patients^9^. In 2021, the incidence of liver injury associated with remdesivir use was 15.2%^46^. Remdesivir adds an additional risk of liver impairment for the patients infected with SARS-CoV-2, as its hepatotoxicity has been proven - it reversibly increases liver enzymes^44^. Therefore, it is not recommended in pediatric patients with baseline ALT 5 times higher than the upper limit of normal. Liver function tests should be performed before starting remdesivir^43^, as well as every day during treatment^4^.

Drug interactions should be taken into consideration due to the risk of increased hepatotoxicity imposed by associating remdesivir intake with P-glycoprotein inhibitors such as hydroxychloroquine, azithromycin, cyclosporine or tacrolimus^43^.

### 6.3 Cardiovascular adverse drug reactions

Acute coronary syndrome, acute heart failure, arrhythmias and myocarditis are some of the cardiovascular afflictions caused by COVID-19^9^. However, remdesivir could also be responsible for some cardiac adverse drug effects: the most talked about in literature is reversible sinus bradycardia, found in both adult and pediatric patients^33^, but there is also proof that it could account for cardiac arrest, shock, hypotension, atrial fibrillation, ventricular tachycardia or ventricular fibrillation, probably through decreasing the cell viability of human pluripotent stem cell cardiomyocytes^47^. Some speculations have been made regarding the mechanisms that are involved in producing bradycardia: because remdesivir is an adenosine analog, it could block the atrioventricular node, or it could bind to human mitochondrial RNA polymerase, producing cardiotoxicity^35^.

All COVID-19 pediatric patients receiving remdesivir should be monitored through electrocardiography^4^.

### *6.4* Other adverse drug events 

Other adverse drug effects cited by studies are hypersensitivity reactions (including variations in blood pressure and heart rate), labial and periocular edema, rash, nausea, hypoxemia, wheezing, shortness of breath, fever, sweating or shivering^6^.

## 7. Benefits and Limitations

While positive effects of remdesivir have been ascertained through various studies, controversy has surrounded remdesivir since its initial FDA approval in 2020.

Remdesivir has proven itself beneficial in outpatient care, reducing hospitalization and mortality by 87% (if administered early after COVID-19 diagnosis)^48^. Another study in favor of remdesivir administration is the double-blind, randomized Adaptive COVID-19 Treatment Trial (ACTT-1), which has also shown reduced hospitalization (10 days, compared to 15 days in patients who received placebo)^49^. It is also thought to be responsible for reducing the time of mechanical ventilation or extracorporeal membrane oxygenation in severe COVID-19 cases^1^.

However, several studies have shown contradictory results, some of them stating that remdesivir has no impact whatsoever on treating SARS-CoV2-infection. The most important is the Solidarity trial conducted by the World Health Organization (WHO) in 2020. It involves 405 hospitals from 30 countries, and it has concluded that remdesivir does not reduce COVID-19 mortality and length of hospital stay. The timing of the FDA approval was debated as there had also been a smaller study previously published in April 2020 stating the absence of benefits in remdesivir treatment, with no impact on SARS-CoV-2 viral load, that had not been taken into consideration by FDA within remdesivir approval^50^. Another study made on a large group of hospitalized COVID-19 veterans treated with remdesivir has actually found an increased length of hospital, without significantly improving survival^49^.

Remdesivir is actually contraindicated in patients with multiorgan dysfunction, vasopressor support, co-administration of other antivirals, elevated ALT or severe renal impairment^19^, as liver and kidney adverse drug effects have been stated in remdesivir-treated COVID-19 patients^50^. It is also not recommended for patients suffering from MIS-C^12^.

Studies have shown that remdesivir penetration into lung tissue might not reach optimal tissue levels in all patients leading to variable therapeutic response^51^, which might also be caused by different timing of administration. Its best effects were obtained during high viral load (first 5 days of symptoms), while administration in advanced stages has shown poor efficiency^52^.

A disadvantage of remdesivir therapy is that currently it can only be administered intravenously. However, developing an orally bioavailable variant is certainly a plausible scenario for the future^48^.

There are also limitations involving drug interactions, as remdesivir is both a substrate for some cytochromes (CYP2C8, CYP2D6, CYP3A4) in vitro, and hydrolase, which is responsible for its metabolism^12^. Hydroxychloroquine and remdesivir should not be administered simultaneously because of the antagonistic effects that the former exerts on the latter^24^.

## 8. Conclusion

Since SARS-CoV-2 can affect the entire pediatric population, with different degrees of severity that range from asymptomatic to lethal, finding efficient forms of treatment is seen as a priority. While most of the studies regarding COVID-19 therapeutic measures are conducted in the adult population, many studies and case reports showing good results of remdesivir use in children have emerged. The most influential nowadays is the CARAVAN cohort study, which is currently in progression (with an estimated primary completion date in February 2023).

However, while the future appears to be brighter for young children infected with SARS-CoV-2 due to the new FDA approval of remdesivir for treating COVID-19, the long-term and short-term effects of this drug should continuously be tracked and investigated, since not all the studies have suggested a positive outcome regarding its efficiency. Thus, its administration still remains controversial and it is undoubtedly not a replacement for the prophylactic measures, such as vaccination.
